# Supplementary figures and images for: Inhibition of CCl4-induced liver inflammation and fibrosis by a NEU3 inhibitor
Source: PLoS One. 2024 Nov 21;19(11):e0308060. doi: 10.1371/journal.pone.0308060 (PMC11581222; doi:10.1371/journal.pone.0308060)

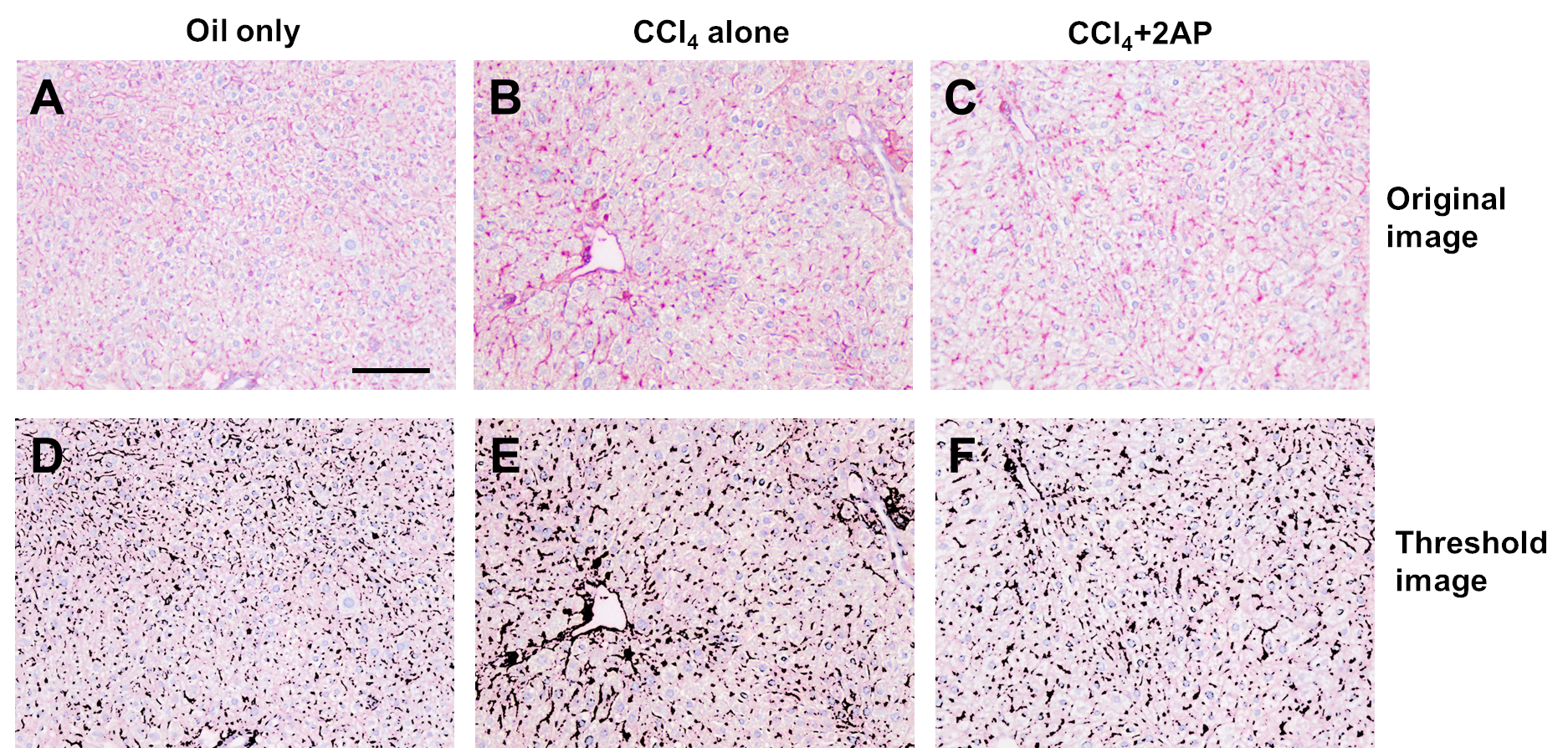

Supplement: S1 Fig — A-C) Representative liver sections were stained with PNA lectin (red staining). Bar is 0.1 mm. D-F) Threshold masking (black) of lectin-stained areas used for quantification. (TIF) [file pone.0308060.s001.tif]

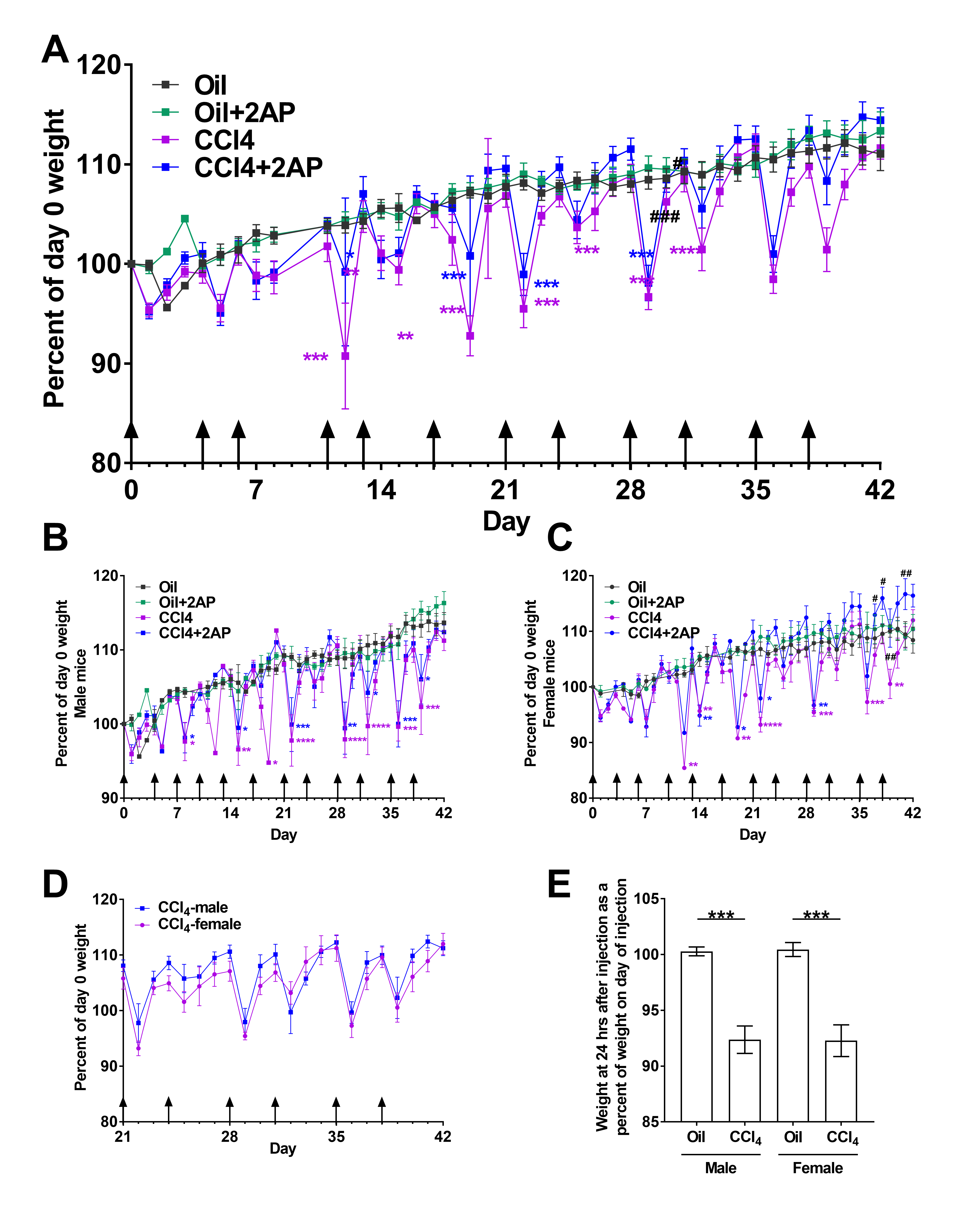

Supplement: S2 Fig — C57BL/6 male and female mice received injections of oil or CCl4 in oil twice a week for 42 days. Starting at day 21, mice also received daily injections of 2AP or buffer control. Mice were euthanized at day 42. Graphs show body weights of A) male and female mice combined, B) male mice only, and C) female mice only. D) Comparison of body weight changes after CCl4 injections in male and female mice from days 21 to 42. E) Percent weight change at 24 hours after injection of oil or CCl4 in oil as a percent of weight on day of injection in male and female mice. Values are mean ± SEM, n = 3–6 mice per group. * indicates p < 0.05, **p < 0.01, ***p < 0.001, ****p < 0.0001 comparing control mice to mice on CCl4 alone, or CCl4+2AP (two-way ANOVA, Dunnett’s test). # indicates p < 0.05, ## p< 0.01, and ### p<0.001 comparing mice on CCl4 alone with CCl4+2AP (two-way ANOVA, Dunnett’s test). E) *** p < 0.001 (one-way ANOVA, Dunnett’s test). Arrows indicate days when mice received injections of oil or CCl4. (TIF) [file pone.0308060.s002.tif]

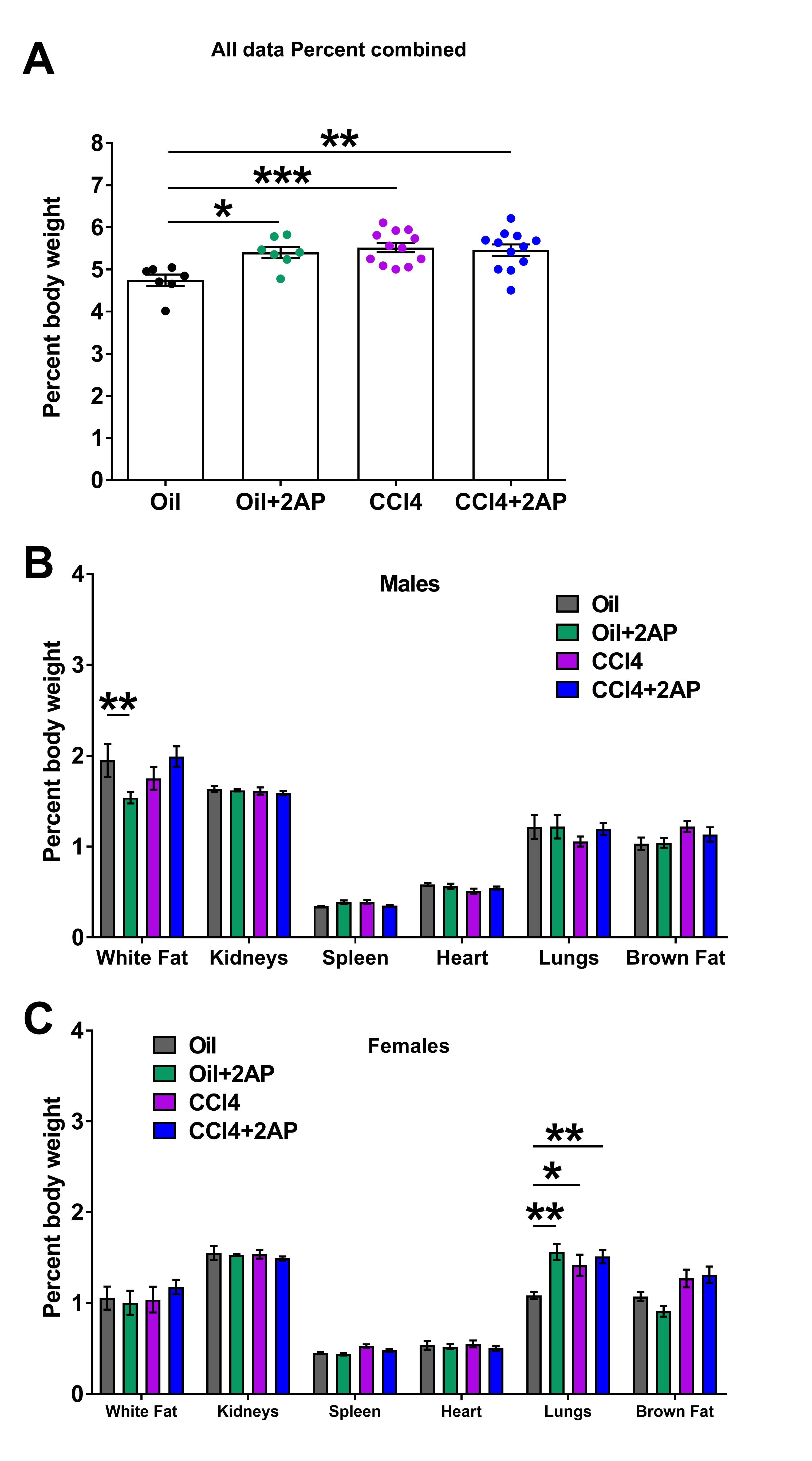

Supplement: S3 Fig — C57BL/6 mice were treated as in Fig 1 and euthanized at day 42. A) Liver weights from male and female mice combined, B) organ weights from male mice only, and C) organ weights from female mice only. Values are mean ± SEM, n = 3–6 mice per group. * p < 0.05, **p < 0.01, ***p < 0.001 (one-way ANOVA, Dunnett’s test). (TIF) [file pone.0308060.s003.tif]

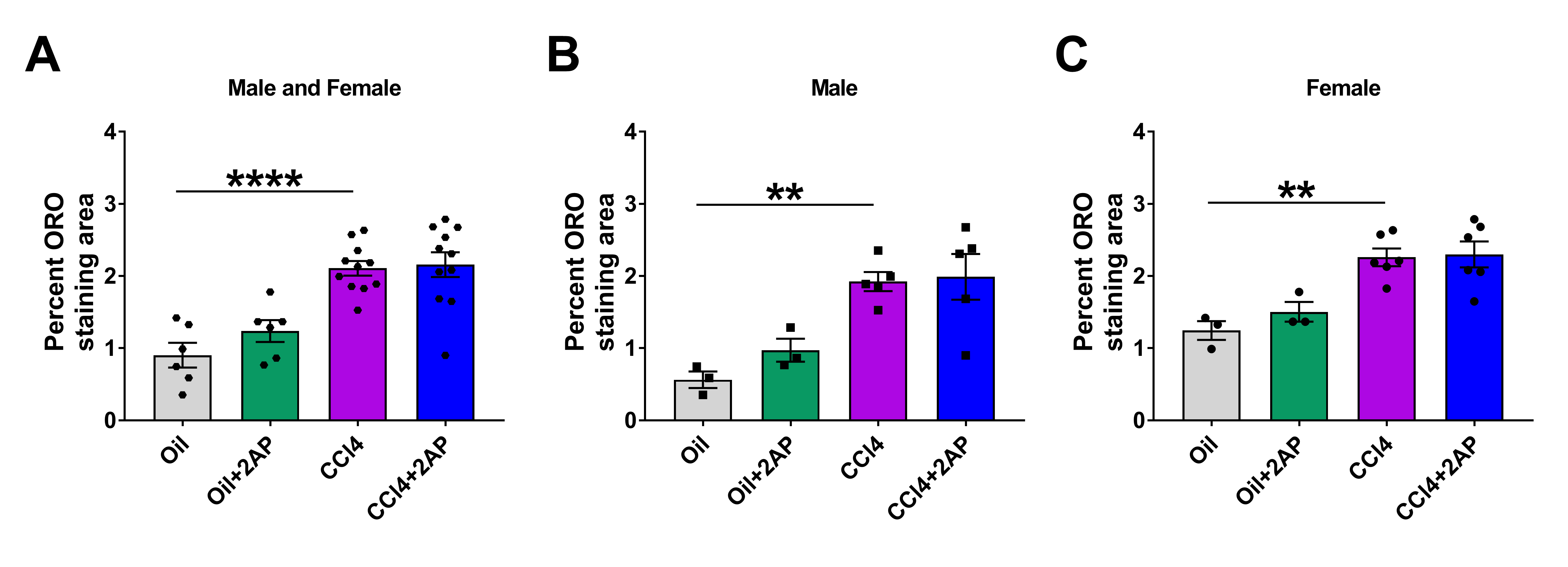

Supplement: S4 Fig — A-C) Quantification of liver sections stained with oil red O (ORO). Values are mean ± SEM, n = 3 to 6 mice per group. **p < 0.01 and ****p < 0.0001 (one-way ANOVA, Dunnett’s test). (TIF) [file pone.0308060.s004.tif]

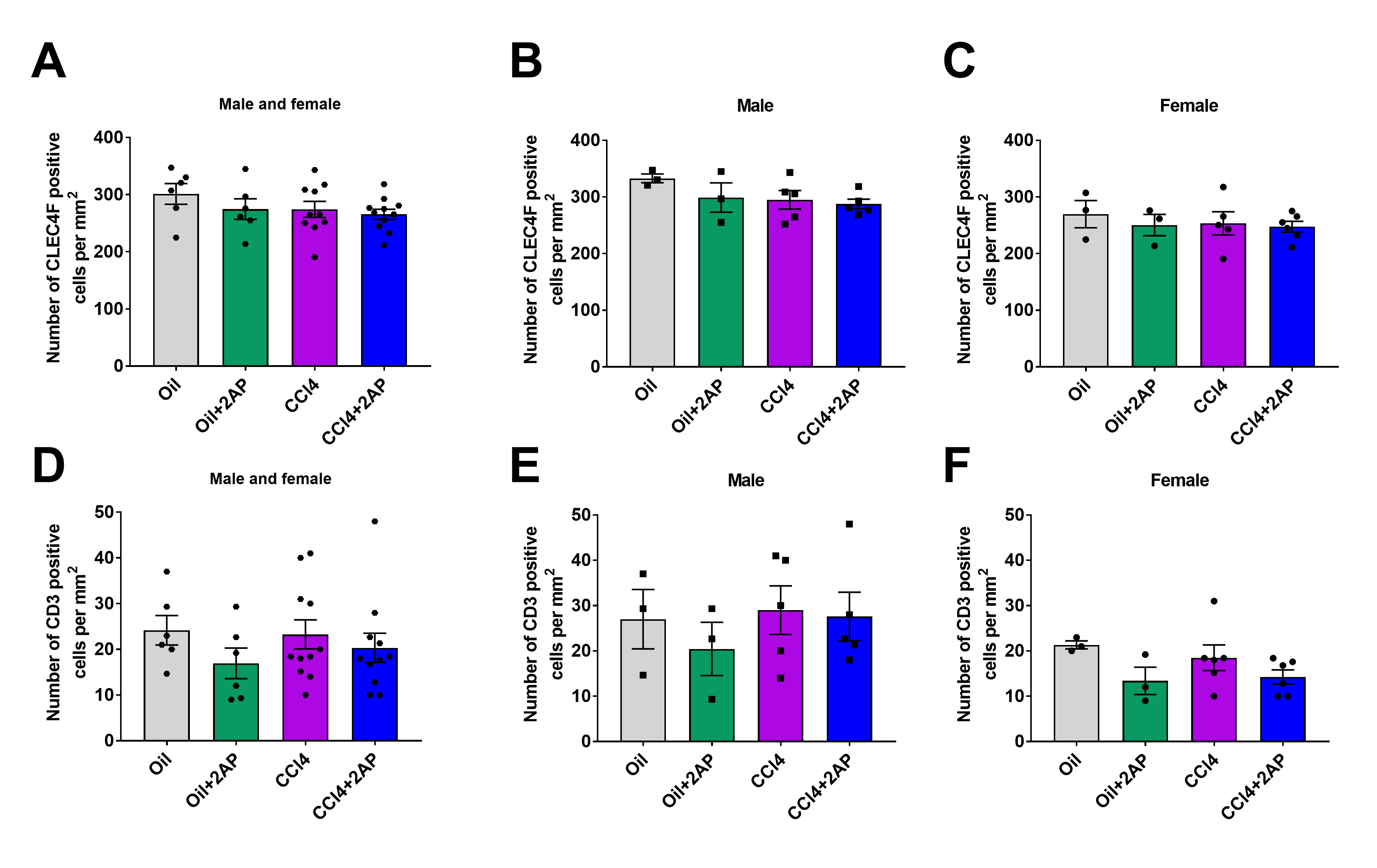

Supplement: S5 Fig — Quantification of liver sections stained with antibodies for A-C) CLEC4F-positive Kupffer cells, D-F) CD3-positive T cells. Values are mean ± SEM, n = 3 to 6 mice per group. (TIF) [file pone.0308060.s005.tif]

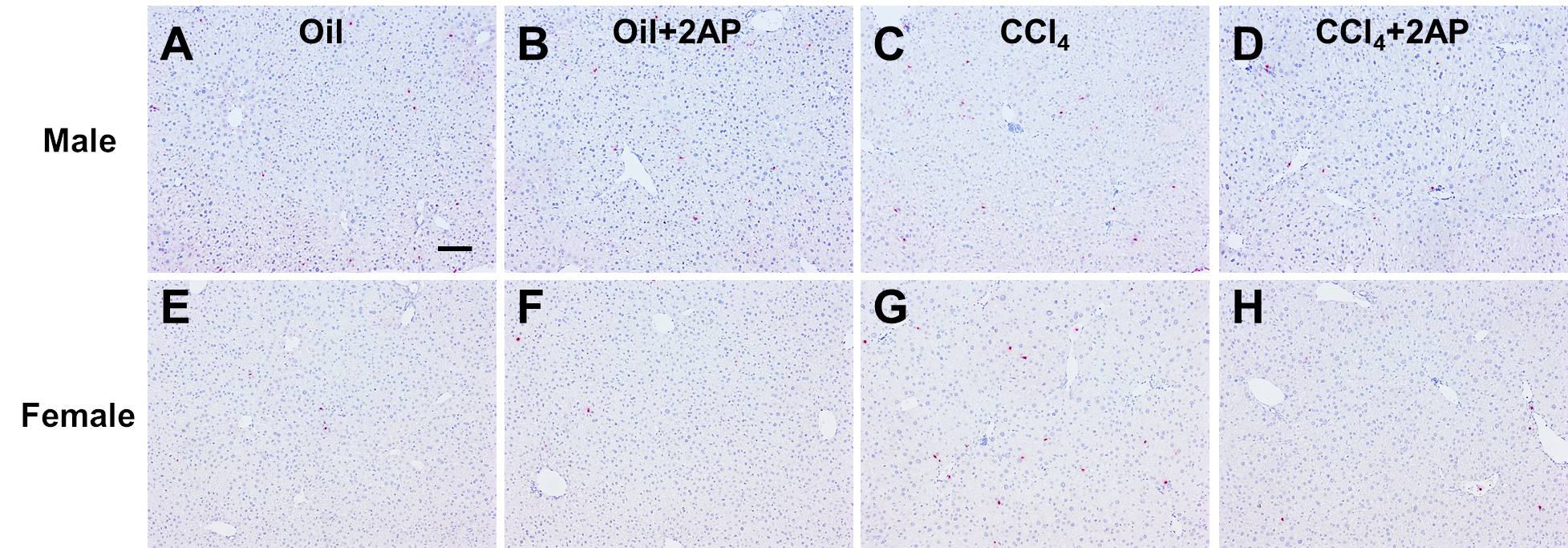

Supplement: S6 Fig — Representative liver sections of A-D) male and E-H) female mice were stained with anti- MRP8 antibodies. Bar is 0.1 mm. (TIF) [file pone.0308060.s006.tif]

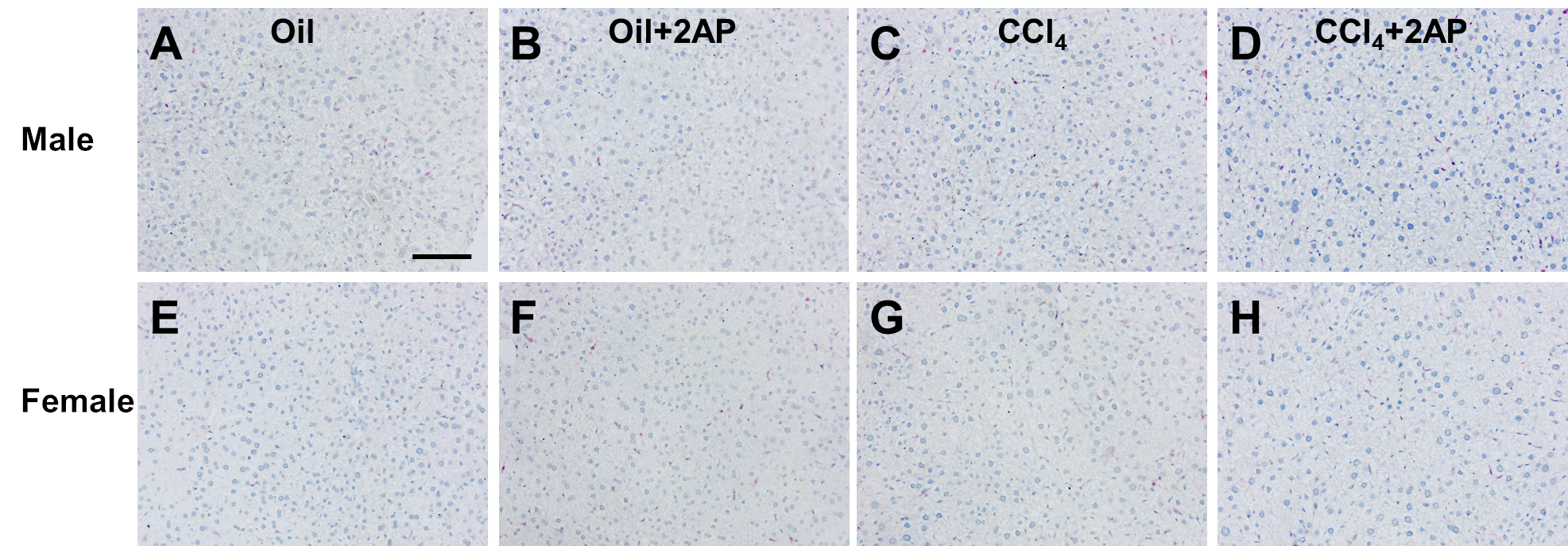

Supplement: S7 Fig — Representative liver parenchyma sections of A-D) male and E-H) female mice were stained with anti-Mac2 antibodies. Bar is 0.1 mm. (TIF) [file pone.0308060.s007.tif]

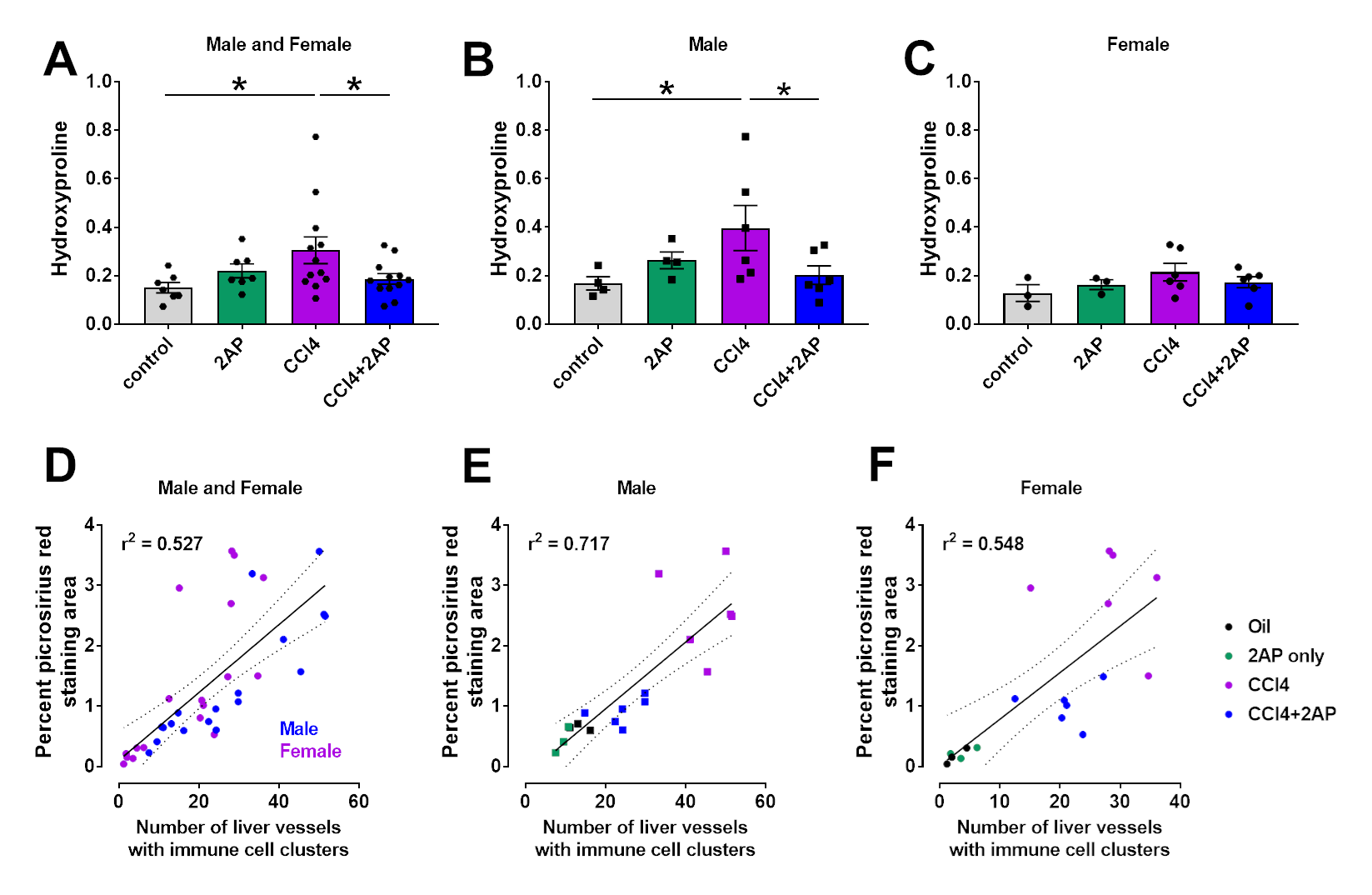

Supplement: S8 Fig — A-C) Quantification of liver tissue for hydroxyproline (μg/mg liver tissue). Values are mean ± SEM, n = 3 to 6 mice per group. *p < 0.05 (one-way ANOVA, Dunnett’s test). D-F) Correlation of the number of liver blood vessels with immune cell clusters with percent picrosirius red staining. (TIF) [file pone.0308060.s008.tif]

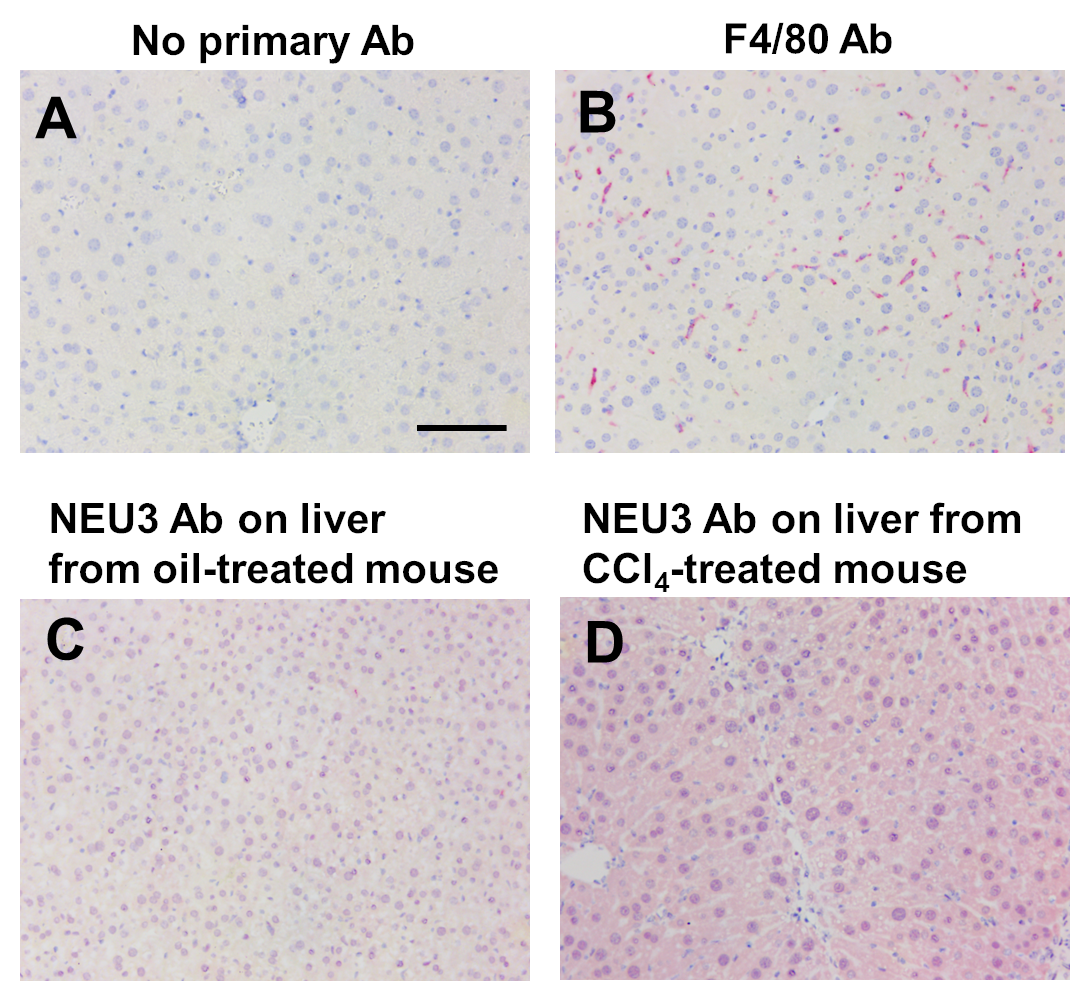

Supplement: S9 Fig — Sections of livers from A-C) oil alone control or D) CCl4 injected mice were stained with A) no primary antibodies, B) anti-F4/80 antibodies, or C) and D) anti-NEU3 antibodies. Bar is 0.1 mm. (TIF) [file pone.0308060.s009.tif]

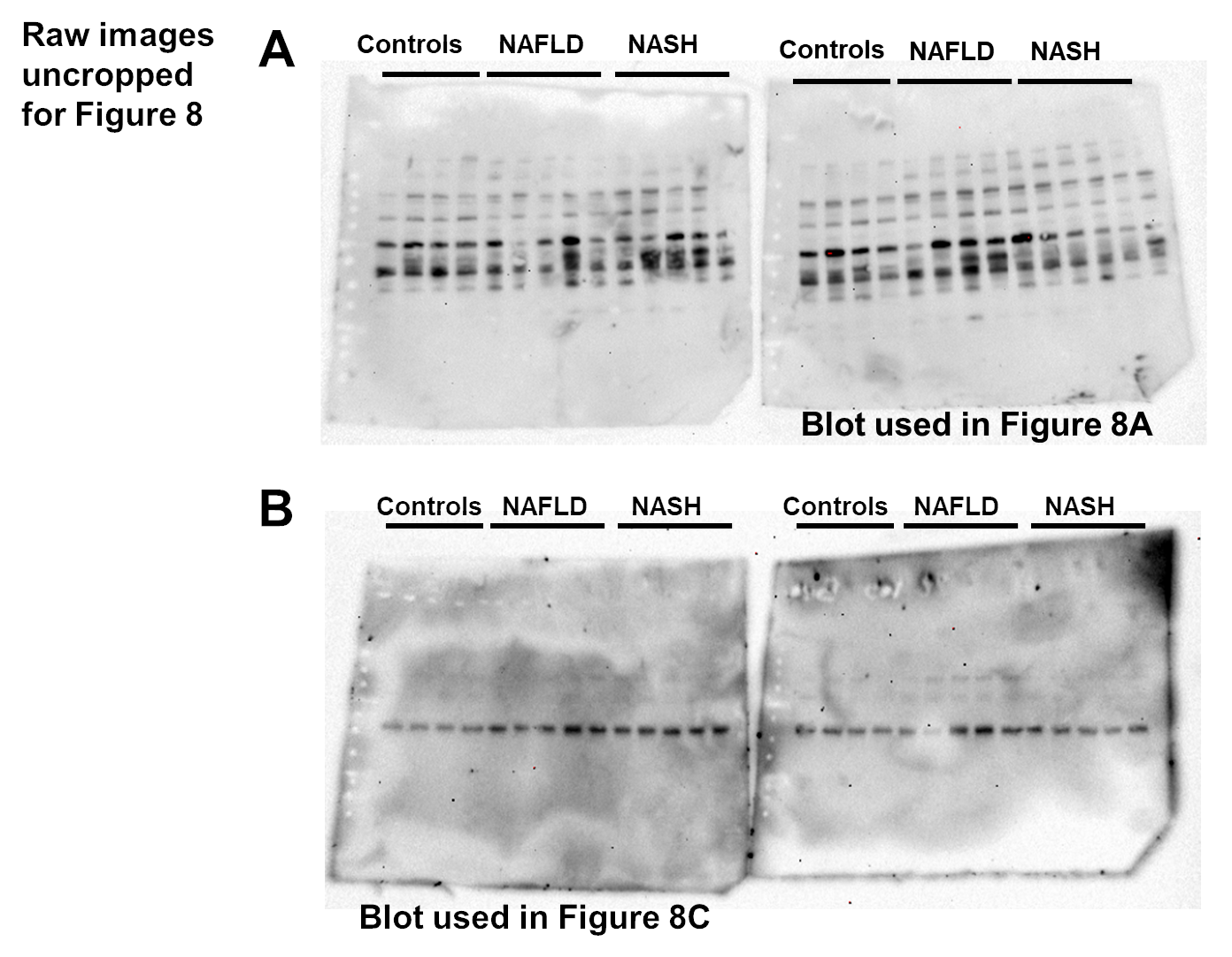

Supplement: S1 Raw image — “Sera from NAFLD patients show elevated desialylation of a glycoprotein and elevated levels of NEU3”. A) Whole raw blot image of western blots of serum samples from young and old healthy controls, NAFLD patients, and NASH patients was stained with RCA lectin. B) Whole raw blot images of western blots of human serum samples was stained with anti-NEU3 antibodies. (TIF) [file pone.0308060.s013.tif]
