# Supplementary material for: Inhibition of CCl4-induced liver inflammation and fibrosis by a NEU3 inhibitor
Source: PLoS One. 2024 Nov 21;19(11):e0308060. doi: 10.1371/journal.pone.0308060 (PMC11581222; doi:10.1371/journal.pone.0308060)
Supplement: S1 Table — Clinical data from the National Institute of Diabetes and Digestive and Kidney Diseases Central Repository (NIDDK-CR). (DOCX) [file pone.0308060.s011.docx]

**Table S1: Clinical Details of patients**

|  | Sex | Age - years  (mean ± SD | ALKA - U/L  (mean ± SEM) | GGT - U/L  (mean ± SEM) | Steatosis  >34% | Any  Inflamm. | Lobular  Inflamm.  0 v 1 | Portal  Inflamm. | NAS >5 |
| --- | --- | --- | --- | --- | --- | --- | --- | --- | --- |
| Young Controls | 2 male  2 female | 22 ± 3.4 | nd | nd | nd | nd | nd | nd | nd |
| Older controls | 2 male  2 female | 62.25 ± 2.9 | nd | nd | nd | nd | nd | nd | nd |
| NAFLD | 5 male  5 female | 50.3 ± 9.8 | 64.1 ± 3.7 | 40.5 ± 11.5 | 0.4 ± 0.2 | 0.3 ± 0.15 | 0.2 ± 0.1 | 0 ± 0 | 0.2 ± 0.13 |
| NASH | 5 male  5 female | 56.9 ± 8.5 | 89.5 ± 13.6 | 78.3 ± 13.6 | 0.5 ± 0.2 | 1.0 ± 0.0 | 0.6 ± 0.2 | 0.4 ± 0.16 | 0.7 ± 0.15 |
| t-test  (NAFLD v NASH) | ns | ns | p = 0.0158 | p = 0.048 | ns | p = 0.003 | ns | p = 0.025 | p = 0.024 |

Clinical data from the National Institute of Diabetes and Digestive and Kidney Diseases Central Repository (NIDDK-CR) used in Figure 7. Non-alcoholic fatty liver disease (NAFLD, now MASLD) and non-alcoholic steatohepatitis (NASH, now MASH) were defined by NIDDK Nonalcoholic Steatohepatitis Clinical Research Network (NASH CRN) working group [1]. Liver function test - Alkaline phosphatase (ALKA), Gamma-glutamyltransferase (GGT). Steatosis, general liver inflammation (Any Inflamm.), lobular inflammation (Lobular Inflamm.), portal inflammation (Portal Inflamm.), and NAFLD Activity Score (NAS) score >5 were scored as 0 v 1. Values are mean ± SEM.

nd – not determined. ns – not significant.

1. Neuschwander-Tetri, B.A., et al., *Clinical, laboratory and histological associations in adults with nonalcoholic fatty liver disease.* Hepatology, 2010. **52**(3): p. 913-24.
