# Supplementary material for: Inhibition of CCl4-induced liver inflammation and fibrosis by a NEU3 inhibitor
Source: PLoS One. 2024 Nov 21;19(11):e0308060. doi: 10.1371/journal.pone.0308060 (PMC11581222; doi:10.1371/journal.pone.0308060)
Supplement: S2 Table — CYP450 enzymes and a panel of receptors were assessed for inhibition by 10 μM 2AP. (DOCX) [file pone.0308060.s012.docx]

**Table S2: Eurofins CYP450 and receptor inhibition by 2AP**

| **CYP450 Enzyme** | **%**  **Inhibition** | **Receptor** | **%**  **Inhibition** | **Receptor** | **%**  **Inhibition** |
| --- | --- | --- | --- | --- | --- |
|  |  |  |  |  |  |
| Aromatase / CYP19A1 | 1 | Adenosine A1 | 10 | Histamine H_1_ | 8 |
| CYP1A2 | 3 | Adenosine A2A | 0 | Imidazoline I_2_, Central | 3 |
| CYP2B6 | 0 | Adrenergic α1A | 5 | Muscarinic M_2_ | 4 |
| CYP2C19 | 3 | Adrenergic α1B | 2 | Muscarinic M_3_ | 1 |
| CYP2C8 | 0 | Adrenergic α2A | -5 | Nicotinic Acetylcholine α1, Bungarotoxin | 5 |
| CYP2C9 | 1 | Adrenergic β1 | 3 | Nicotinic Acetylcholine α3β4 | -2 |
| CYP2D6 | 2 | Adrenergic β2 | 5 | Norepinephrine (NET) | 7 |
| CYP3A4 | -2 | Calcium Channel  L-Type, Dihydropyridine | 7 | Opiate μ (OP3, MOP) | 0 |
|  |  | Cannabinoid CB1 | -1 | Potassium Channel [KATP] | -11 |
|  |  | Dopamine D1 | -5 | Potassium Channel hERG | 4 |
|  |  | Dopamine D2S | -4 | Prostanoid EP_4_ | 7 |
|  |  | GABAA Flunitrazepam | -2 | Serotonin  (5-Hydroxytryptamine)  5-HT2_B_ | 12 |
|  |  | GABAA  Muscimol | 4 | Sigma σ1 | 13 |
|  |  | Glutamate | 27 | Sodium Channel, Site 2 | 13 |

Assays were performed as described

(<https://www.eurofinsdiscovery.com/catalog/hitprofilingscreen-cyp450-leadhunter-panel-tw/PP115>).
